# Towards Energy-Aware Feedback Planning for Long-Range Autonomous Underwater Vehicles

**DOI:** 10.3389/frobt.2021.621820

**Published:** 2021-03-19

**Authors:** Tauhidul Alam, Abdullah Al Redwan Newaz, Leonardo Bobadilla, Wesam H. Alsabban, Ryan N. Smith, Ali Karimoddini

**Affiliations:** ^1^ Department of Computer Science, Louisiana State University, Shreveport, LA, United States; ^2^ Department of Electrical and Computer Engineering, North Carolina A&T State University, Greensboro, NC, United States; ^3^ School of Computing and Information Sciences, Florida International University, Miami, FL, United States; ^4^ College of Computer and Information Systems, Umm Al-Qura University, Makkah, Saudi Arabia; ^5^ Institute of Environment, Florida International University, Miami, FL, United States

**Keywords:** feedback planning, energy-aware, long-range autonomous underwater vehicles, predictive ocean model, kinematic model, state uncertainty model

## Abstract

Ocean ecosystems have spatiotemporal variability and dynamic complexity that require a long-term deployment of an autonomous underwater vehicle for data collection. A new generation of long-range autonomous underwater vehicles (LRAUVs), such as the Slocum glider and Tethys-class AUV, has emerged with high endurance, long-range, and energy-aware capabilities. These new vehicles provide an effective solution to study different oceanic phenomena across multiple spatial and temporal scales. For these vehicles, the ocean environment has forces and moments from changing water currents which are generally on the order of magnitude of the operational vehicle velocity. Therefore, it is not practical to generate a simple trajectory from an initial location to a goal location in an uncertain ocean, as the vehicle can deviate significantly from the prescribed trajectory due to disturbances resulted from water currents. Since state estimation remains challenging in underwater conditions, feedback planning must incorporate state uncertainty that can be framed into a stochastic energy-aware path planning problem. This article presents an energy-aware feedback planning method for an LRAUV utilizing its kinematic model in an underwater environment under motion and sensor uncertainties. Our method uses ocean dynamics from a predictive ocean model to understand the water flow pattern and introduces a goal-constrained belief space to make the feedback plan synthesis computationally tractable. Energy-aware feedback plans for different water current layers are synthesized through sampling and ocean dynamics. The synthesized feedback plans provide strategies for the vehicle that drive it from an environment’s initial location toward the goal location. We validate our method through extensive simulations involving the Tethys vehicle’s kinematic model and incorporating actual ocean model prediction data.

## 1 Introduction

Ocean ecosystems are complex and have high variability in both time and space. Consequently, ocean scientists must collect data over long periods to obtain a synoptic view of ocean ecosystems and understand their spatiotemporal variability. To support data collection, autonomous underwater vehicles (AUVs) are increasingly being used for studying different oceanic phenomena such as oil spill mapping (Kinsey et al., 2011), harmful algal blooms (Das et al., 2010), phytoplankton and zooplankton communities (Kalmbach et al., 2017), and coral bleaching (Manderson et al., 2017). These AUVs can be classified into two categories: 1) propeller-driven vehicles, such as the Dorado class, which can move fast and gather numerous sensor observations but are limited in deployment time to multiple hours; and 2) minimally-actuated vehicles such as drifters, profiling floats, and gliders that move slower, but can remain on deployment for tens of days to multiple weeks.

**FIGURE 1:**
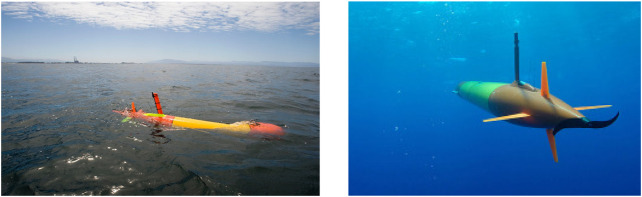
Two instances of a Tethys-class vehicle deployed in the ocean (MBARI, 2009).

A new generation of the long-range autonomous underwater vehicles (LRAUVs), i.e., Tethys, combines the advantages of both minimally-actuated and propeller-driven AUVs (Hobson et al., 2012). These LRAUVs can move quickly for hundreds of kilometers, float with water currents, and carry a broad range of data collection sensors. They can also control their buoyancy for changing depths in the water and the angle at which they move through the water. By mixing modalities, an LRAUV can be deployed in the water for weeks at a time and navigate challenging ocean current conditions for long periods. Two instances of deployed Tethys AUVs are shown in Figure 1. A planning and control technique for this vehicle is critical to increase its autonomy and generate mission trajectories during long-range operations. The execution of a planned trajectory for this vehicle is also challenging due to ocean currents’ variability and uncertainty. Thus, it is not practical to generate a simple navigation trajectory from an initial location to a goal location in a dynamic ocean environment because the vehicle can deviate from its trajectory due to motion noise and cannot estimate its state accurately in underwater environments due to sensor noise.

To address these challenges, we consider the use of feedback motion planning for an LRAUV by combining its kinematic modeling and an ocean dynamic model while also incorporating motion and sensor uncertainties. A feedback plan is calculated over each ocean current layer in an underwater environment for a vehicle inspired by our previous work (Alam et al., 2020) so that the vehicle can adapt its trajectory from any deviated state in the presence of any noise or modeling errors. Furthermore, this feedback plan is crucial when the vehicle state is not fully observable from sensor readings. For such vehicles with partially observable states, a Partially Observable Markov Decision Process (POMDP) provides a standard mathematical model for vehicle motion planning under uncertainties. The two major factors make solving our problem particularly difficult: 1) for the POMDP formulation, finding the optimal solution is formally hard (NP-hard or PSPACE-hard), and 2) our objective is to compute stochastic energy-aware feedback plans using ocean dynamics in contrast to other prior POMDP feedback planning methods that calculate the stochastic shortest path. A large body of existing research focuses on the stochastic shortest path problem without considering energy constraints. However, it may be unrealistic to assume that the vehicle has unlimited resources in many applications. A more realistic model would consider that an autonomous vehicle has limited stored energy, which continually depletes as it operates. Here, we address this constraint and propose an extension to the POMDP framework that includes energy awareness. Although energy awareness should take into account an initial energy condition, the efficiency of actuation, and the drag effect, our method mostly utilizes ocean currents in our calculations.

### Contributions

In this article, we present a method to synthesize feedback plans for an LRAUV in an underwater environment under motion and sensor uncertainties. First, we develop an ocean dynamic model from ocean current prediction data. Second, a goal-constrained belief space is introduced to make the feedback plan synthesis computationally tractable. Finally, energy-aware feedback plans for several water current layers are synthesized by utilizing sampling and the ocean dynamic model.

A preliminary version of this article appeared in (Orioke et al., 2019). This article is fundamentally different in that it extends (Orioke et al., 2019) by incorporating motion uncertainty and sensor uncertainty coupled with energy awareness from the water flow of an underwater environment within a modified POMDP framework.

## 2 Related Work

The feedback mission control of autonomous underwater vehicles in dynamic and spatiotemporal aquatic environments has attracted a great deal of interest. A feedback trajectory tracking scheme was developed for an AUV in a dynamic oceanic environment with modeled and unmodeled uncertainties (Sanyal and Chyba, 2009). An informative feedback plan was generated for AUVs to visit essential locations by estimating Kriging errors from spatiotemporal fields (Reis et al., 2018). An obstacle avoidance method (Kawano, 2006) is presented, where an MDP-based re-planner considers only the geometrical properties of obstacles and the dynamics and kinematics of an AUV to find and track its target path. An adaptive mission plan for an AUV according to its available resources, such as battery and memory usage, is proposed to add or remove locations for data collection tasks in underwater environments (Harris and Dearden, 2012).

A finite-state automata-based supervisory feedback control (Xu and Feng, 2009) is presented for obstacle avoidance by an AUV. A temporal plan is calculated in (Cashmore et al., 2014) for AUV mission control that optimizes the time taken to complete a single inspection tour. A feedback and replanning framework (Cashmore et al., 2014) is integrated along with the temporal plan in the Robot Operating System (ROS). Sampling Based Model Predictive Control (SBMPC) (Caldwell et al., 2010) is utilized to simultaneously generate control inputs and feasible trajectories for an AUV in the presence of nonlinear constraints.

Open-loop trajectory design methods (Chyba et al., 2009; Smith et al., 2010) drive an AUV from a given initial location to the desired goal location, minimizing a cost in terms of energy and time taken by the vehicle. The implementation of open-loop trajectories for AUVs works well in environments without any model uncertainties. In our previous work (Alam et al., 2018a, 2020), we have proposed an open-loop approach for solving the problem of deploying a set of minimally-actuated drifters for persistent monitoring of an aquatic environment. In our another work (Alam et al., 2018b), we predicted the localized trajectory of a drifter for a sequence of compass observations during its deployment in a marine environment. We presented a closed-loop approach (Alam et al., 2018b) when an AUV has a considerable unpredictability of executing its action in a dynamic marine environment. Moreover, the previous studies (Bellingham et al., 2010; Hobson et al., 2012) on the Tethys AUV described the mission and other capabilities of the vehicle. However, there is no work on the development of a planning algorithm for controlling the vehicle.

Various types of rewards modification in POMDPs have been investigated in previous research efforts (Lee et al., 2018; Kim et al., 2019). Typically, the reward function in POMDPs is designed to solve the stochastic shortest path problem, where the goal is to compute a feedback plan that reaches a target state from a known initial state by maximizing the expected total reward. From a motion planning point of view, the reward can be replaced by a cost, where the goal is to minimize the expected total cost. In both cases, the sequence of rewards or costs, however, can be aggregated by considering the discounted reward (cost) or the average reward (cost).

A point-based algorithm to calculate approximate POMDP solutions is presented combining the full and partial observable components of an AUV’s state to reduce the dimension of its belief space (Ong et al., 2009). An efficient point-based POMDP algorithm for AUV navigation (Kurniawati et al., 2008) exploiting the optimally reachable states is developed to improve computational efficiency. A point-based POMDP approach (Kurniawati and Patrikalakis, 2013) is presented, where the original solution is updated by modifying a set of sample beliefs. The planning for hydrothermal vent mapping problems using information from plume detections is modeled as a POMDP utilizing the reachable states as the current state of an AUV (Saigol et al., 2009). In this work, an information likelihood algorithm is proposed turning the POMDP into an information state MDP. An online POMDP solver (Kurniawati and Yadav, 2016) based on an adaptive belief tree is proposed to improve the existing solution and update the solution when replanning is needed in dynamic environments.

To the best of our knowledge, this is the first work for synthesizing energy-aware feedback plans from a POMDP solution for an underwater vehicle using water flow under motion and sensor uncertainties. In our work, we utilize an LRAUV’s sensor readings to control its mission operation, taking into account its several drifting and actuation capabilities.

## 3 Preliminaries

In this section, we describe a representation of an underwater environment and motion and observation (sensing) models for our vehicle with relevant definitions. Then, we formulate our problem of interest.

First, we consider a 3-D environment where a workspace is an ocean environment denoted as $W\subset {\mathbb{R}}^{3}$. The workspace is divided into a set of 2-D water current layers at different depths of the environment which are represented by the third dimension. Let *L* be the total number of water current layers in the environment.

Definition 3.1 (Workspace). The workspace is defined as $W=W_{1}\cup W_{2}\cup \cdots \cup W_{L}$. At each current layer, we model the workspace $W_{l}\subset {\mathbb{R}}^{2}$, where l∈{1,…,L}, as a polygonal environment. Let $O_{l}\subset {\mathbb{R}}^{2}$ be the land and littoral region of the environment at each layer which is considered an inaccessible region for the vehicle. The free water space at each current layer is composed of all navigable locations for the vehicle, and it is defined as $E_{l}=W_{l}\setminus O_{l}$. The free water space in the whole workspace is denoted by $ℰ=E_{1}\cup E_{2}\cup \cdots \cup E_{L}$. We discretize each workspace layer $W_{l}$ as a 2-D grid. Each grid point or location, denoted as q, has a geographic coordinate in the form of longitude, latitude, and depth (water current layer) q=(x,y,l), where x,y∈ℝ and l∈{1,…,L}.

Second, in our vehicle motion model, we incorporate noise and uncertainty in the vehicle’s movement to account for the modeling error and unmodeled dynamics.

Definition 3.2 (Motion Model). The state space for the vehicle is defined as X=ℰ×Θ in which Θ is the set of angles such that θ∈Θ, and θ represents the vehicle’s orientation. At time *t*, the vehicle state in the state space is represented by $x_{t}=\left(x_{t},y_{t},l_{t},\theta_{t}\right)$ in which $\left(x_{t},y_{t},l_{t}\right)$ denotes the vehicle’s position in the free water space, and $\theta_{t}$ provides the vehicle’s orientation.

The motion model f of the vehicle can be written as

$$x_{t+1}=f\left(x_{t},u_{t},d_{t}\right),$$ (1)

where $x_{t}$ is the vehicle state, $d_{t}$ is motion noise, and $u_{t}$ is the action belonging to a set of admissible actions *U* such that $u_{t}\in U$.

Third, it is assumed that our vehicle can observe its positions and the goal location with uncertainties due to imperfect sensor readings and the dynamic nature of an underwater environment.

Definition 3.3 (Observation Model). Let *Y* be the observation space, which is the set of all possible sensor observations y∈Y, the vehicle receives. The observation model h of the vehicle can be represented as below.

$$y_{t}=h\left(x_{t},w_{t}\right),$$ (2)

where $w_{t}$ denotes sensor noise.

It is challenging to plan in an uncertain, stochastic environment when there are motion and observation uncertainties in a vehicle model. To formulate this planning problem, it is necessary to connect hidden states and observations of our vehicle. A generic model in this context is Partially Observable Markov Decision Processes (POMDPs).

Definition 3.4 (POMDP). A POMDP is defined by a tuple P=(X,U,f,R,Y,h,γ), where

• X is a finite set of states.
• U is a finite set of actions, available to the vehicle.
• $f\left(x,u,d,x^{\prime }\right)=p\left(x^{\prime }|x,u,d\right)$ is a probabilistic transition function, which defines the probability of moving to a state $x^{\prime }\in X$ after taking an action u∈U and sustaining a noise d in a state x∈X.
• R(x,u) is a reward function, which defines a real-valued reward after taking an action u∈U in a state x∈X.
• *Y* is a finite set of observations for the vehicle.
• $h\left(x^{\prime },u,y\right)=p\left(y|x^{\prime },u\right)$ is a probabilistic observation function, which defines the probability of observing y∈Y after taking an action u∈U and reaching a state $x^{\prime }\in X$.
• γ∈[0,1) is a discount factor.

Due to sensor noise, observations of our vehicle provide only partial information over the states. Planning with partial information can be framed as a search problem in a belief space. Let *B* be the belief space.

Definition 3.5 (Belief). A belief state $b_{t}\in B$ of the vehicle is defined as a posterior distribution over all possible states given the past actions and sensor observations $b_{t}=\left(x_{t}|u_{0},\ldots ,u_{t-1},y_{0},\ldots ,y_{t}\right)$. The belief state $b_{t}$ can be recursively updated with the following transition function τ (Kim et al., 2019)

$$b_{t}=\tau \left(b_{t-1},u_{t-1},y_{t}\right),$$ (3)

in which the next belief state depends only on the current belief state, action, and observation.

Typically, the POMDP solution can be found by solving the equivalent belief MDP where every belief is a state.

Definition 3.6 (Belief MDP). An equivalent belief MDP is defined by a tuple P=(B,U,τ,R,γ), where

• *B* is the set of belief states over the POMDP states.
• *U* is a finite set of actions, available to the vehicle as for the original POMDP.
• τ is the belief state transition function.
• R(b,u) is the reward function on belief states.
• γ∈[0,1] is a discount factor equivalent to the γ in the original POMDP.

A feedback plan is called a solution to a belief MDP problem if it causes the goal state to be reached from every belief state in *B*. Let $b_{g}\in B$ be a goal belief state of the vehicle at any water current layer of the environment. Our objective of the article is to compute a feedback plan for our vehicle.

Definition 3.7 (Feedback Plan). A feedback plan π is defined as a function over the belief space π:B→U to produce an action π(b)=u∈U, for a belief state b∈B, to reach the goal belief state $b_{g}$.

The value function of a feedback plan π is computed from the expected discounted reward at the current belief state *b* as follows:

$$V_{\pi }\left(b\right)=E\left(\sum_{t=0}^{\infty }\gamma^{t}R\left(b_{t},\pi \left(b_{t}\right)|b_{0}\right)\right),$$ (4)

where γ is the discount factor, and $b_{0}$ is the initial belief state. This value function is maximized for the optimal feedback plan $\pi^{\text{*}}$ as follows:

$$\pi^{*}\left(b\right)=\arg\underset{\pi }{\max}V_{\pi }\left(b\right),\;\forall b\in B.$$ (5)

### 3.1 Problem Formulation

**FIGURE 2:**
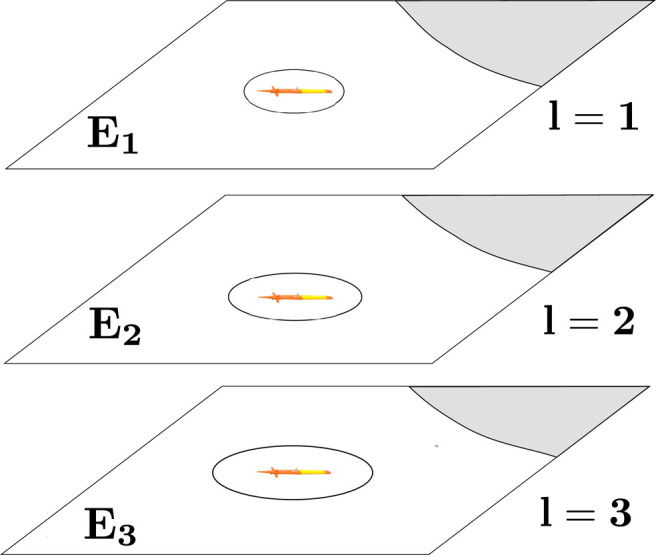
Localization uncertainty of a vehicle increases as it goes down along different water current layers.

In our 3-D workspace W, we account for different localization uncertainties due to sensor noise for its divided 2-D water current layer at different depths. Specifically, we consider an almost reliable localization on the water surface layer (first water current layer) since the GPS information is accessible to the vehicle on the water surface. As the vehicle goes deeper in the water column, its localization uncertainty is assumed to increase due to the implied time increase between potential GPS fixes, as illustrated in Figure 2. In that circumstance, the vehicle’s state is estimated using dead-reckoning only, and the vehicle is required to navigate to the water surface periodically to keep the localization uncertainty tractable. Thus, the localization uncertainty for the vehicle decreases with its upward motion in the water column; it could conceivably *quickly* surface for a GPS fix with minimal time and/or energy consumption.

When the vehicle is uncertain about its state due to sensor noise and has also motion uncertainty, it is crucial to compute a feedback plan that maps every belief state to an action. In computing a feedback plan, we take the environmental water flow into account as an ocean dynamic model. We assume that this ocean dynamic model and the reward function are known *a-priori*. Our reward function is strictly positive, monotonically increasing toward the goal belief state, and additive. Unlike many prior POMDP feedback planning algorithms that compute the stochastic shortest path, our goal is to compute the stochastic energy-aware path using the ocean dynamic model. Due to the curse of dimensionality of the belief space, it is computationally intractable to synthesize feedback plans for multiple water current layers concurrently. Therefore, we assume that a high-level planner provides an intermediate goal at each water current layer. This motivates us to formulate the following problem to synthesize water current layer-wise feedback plans for our vehicle.

Problem Statement: Given an ocean environment ℰ and its dynamic model, the action set of our vehicle U, the vehicle motion model, and a goal belief state $b_{g}$, compute a feedback plan π for each water current layer that drives the vehicle from a belief state b of the environment to reach the goal belief state $b_{g}$ of the same water current layer.

## 4.METHODOLOGY

In this section, we detail an energy-aware feedback planning method that utilizes sampling and the ocean dynamic model for solving the problem formulated in Section 3.

### 4.1 Ocean Dynamic Model

#### 4.1.1 Data Acquisition

**FIGURE 3:**
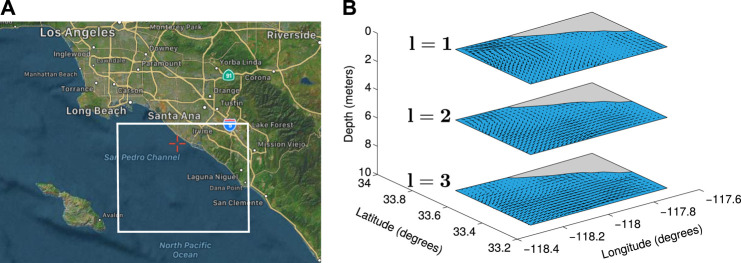
**(A)** The area of interest in the SCB region, California. **(B)** Flow fields generated from ROMS oceanic current prediction data.

We utilize the Regional Ocean Modeling System (ROMS) (Shchepetkin and McWilliams, 2005) predicted oceanic current data in the Southern California Bight (SCB) region, CA, USA, as illustrated in Figure 3A, which is contained within $33^{\circ }17^{\prime }60^{\prime \prime }$ N to $33^{\circ }42^{\prime }$ N and $-117^{\circ }42^{\prime }$ E to $-118^{\circ }15^{\prime }36^{\prime \prime }$ E. ROMS is a free-surface, split-explicit, terrain-following, nested-grid mode, and an extensively used ocean model. ROMS is also an open-source ocean model that is widely accepted and supported throughout the oceanographic and modeling communities. ROMS primarily assimilates surface velocities from HF radar data, and it is assumed that the forecasting for near-surface velocities is accurate in direction and magnitude.

The four dimensions of 4-D ROMS current prediction data are longitude, latitude, depth, and time. The ROMS current prediction data are given at depths from 0 m to 125 m and with 24 h forecast for each day. Each ROMS current velocity prediction is given at depths from 0 m to 4,000 m, with a 12-h hindcast, a 12-h nowcast, and a 48-h forecast each day. The first 24-h comprising hindcasts and nowcasts of each day are the most accurate ocean current prediction in the ROMS model. In our work, we utilize a concatenation of the earliest 24-h of each prediction for each day for 30 days of predictions. The three components of oceanic currents are northing current (*α*), easting current (β), and vertical current (λ). These components are given based on the four dimensions (time, depth, longitude, and latitude).

#### 4.1.2 Water Flow Characterization

We create flow fields at several water current layers from the ROMS ocean current prediction data. Ocean current prediction data for a specific time and at a particular water current layer can be represented as a flow field. Let the flow field on a location *q* at a particular water current layer of the environment $E_{l}$ be F(q). For a location *q* at a particular water current layer, the easting component along the latitude axis is denoted by μ(q), the northing component along the longitude axis is denoted by ν(q), and the vertical component at that water current layer is denoted by ξ(q). The flow field based on two components for a location *q* at that water current layer is specified as:

F(q)=μ(q)i+ν(q)j, (6)

where *i* and *j* are unit vectors along the latitude and longitude axes, respectively.

The vertical component of the ocean current ξ(q) at several water current layers is considered zero. Thus, we create flow fields for three water current layers as illustrated in Figure 3B. Then, we find flow lines of the water flow from these flow fields. Flow lines of the water flow over the flow field *F* are the trajectories or paths traveled by an omnidirectional vehicle at the given water current layer whose vector field is the flow field.

### 4.2 Goal-Constrained Belief Space

**FIGURE 4:**
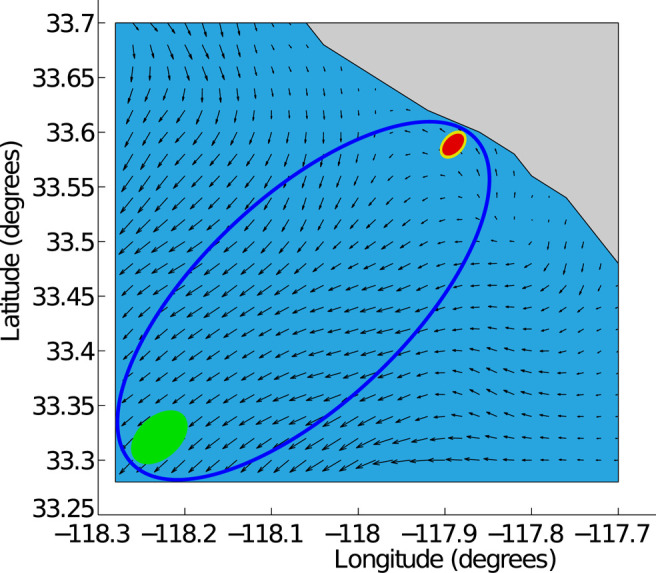
The blue elliptical goal-constrained belief space ${ℛ}^{\text{*}}\left(b_{0},b_{g}\right)$ is given as prior knowledge for the green goal belief state $b_{g}$ from the red initial belief state $b_{0}$ of the vehicle.

It is computationally expensive to compute a feedback plan for a given goal belief state $b_{g}$ of a water current layer under a finite horizon because of the high dimensional belief space *B* (Papadimitriou and Tsitsiklis, 1987). Therefore, we utilize a reachable belief space $ℛ\left(b_{0}\right)$ containing belief states from an initial belief state $b_{0}$ to compute the plan for the water current layer $W_{l}$. The reachable belief space $ℛ\left(b_{0}\right)$ is much smaller than *B* in terms of the number of belief states. Then, we construct a goal-constrained belief space ${ℛ}^{*}\left(b_{0},b_{g}\right)$ containing belief states from an initial belief state $b_{0}$ that drive the AUV to the goal belief state $b_{g}$ of the same water current layer $W_{l}$. The goal-constrained belief space ${ℛ}^{\text{*}}\left(b_{0},b_{g}\right)$ is much smaller than the reachable belief space $ℛ\left(b_{0}\right)$ since ${ℛ}^{\text{*}}\left(b_{0},b_{g}\right)$ is pruned from *B*. This goal-constrained belief space ${ℛ}^{\text{*}}\left(b_{0},b_{g}\right)$ leads to a computationally efficient synthesis of the optimal feedback plan $\pi^{\text{*}}$ for the water current layer $W_{l}$ because any vehicle state sample *x* in $\pi^{\text{*}}$ is taken within ${ℛ}^{\text{*}}\left(b_{0},b_{g}\right)$. The representation of ${ℛ}^{\text{*}}\left(b_{0},b_{g}\right)$ is represented as an ellipse with $x_{0}∼b_{0}$ and $x_{g}∼b_{g}$ as focal points. This ${ℛ}^{\text{*}}\left(b_{0},b_{g}\right)$ can be expressed as

$${ℛ}^{*}\left(b_{0},b_{g}\right)=\left\{b\in B|{\left\|x_{0}-x\right\|}_{2}+{\left\|x_{g}-x\right\|}_{2}<\delta \right\},$$ (7)

where $x_{0}∼b_{0}$, $x_{g}∼b_{g}$, x∼b, and δ is a threshold value which can be tuned to obtain a desired ${ℛ}^{\text{*}}\left(b_{0},b_{g}\right)$. An example ${ℛ}^{\text{*}}\left(b_{0},b_{g}\right)$ is illustrated in Figure 4.

### 4.3 Energy-Aware Feedback Plan Synthesis

We develop our energy-aware feedback planning algorithm based on the Partially Observable Monte Carlo Planning (POMCP) algorithm (Silver and Veness, 2010). The POMCP algorithm assumes that the optimal plan can be synthesized by aggregating rewards of the available actions from each state using the Monte-Carlo Tree Search (MCTS) algorithm. It is an approximate method that does not consider energy awareness, but it is known to extract near-optimal policies in finding the stochastic shortest path where optimal rewards depend on the distance from the goal state. Furthermore, the POMCP algorithm allows us to utilize the domain knowledge. In our work, we use the domain knowledge of the reachable belief space $R^{\text{*}}$ to reduce the search space for choosing actions. Instead of searching actions over all possible events that could happen with low probabilities, the reachable belief space constraints the action search space for the most likely events.

To overcome the challenges associated with solving belief space planning, we first define a set of discrete actions and a set of discrete outcomes. For an LRAUV planning to reach a goal location, we consider nine actions that include actions toward eight compass directions, *i.e.,* N, NE, E, SE, S, SW, W, NW along with drift (idle). The outcomes of actions could be three observations, *i.e.,* goal, intermediate, and outside. In other words, the goal observation refers to the vehicle reaches to the goal location, the intermediate observation refers to it moves toward the goal location, and the outside observation refers to it goes beyond the goal-constrained belief space. Since the outcome of any action is not deterministic, the LRAUV must consider all three observations when simulating an action. For a given state *x*, Algorithm 1 provides a set of preferred actions A based on the goal-constrained belief state. Algorithm 2 returns the optimal feedback plan $\pi^{\text{*}}$ for a water current layer from a history of belief states.

**Algorithm 1:** Preferred_Action ($h,x,U,F,{ℛ}^{\text{*}}$).
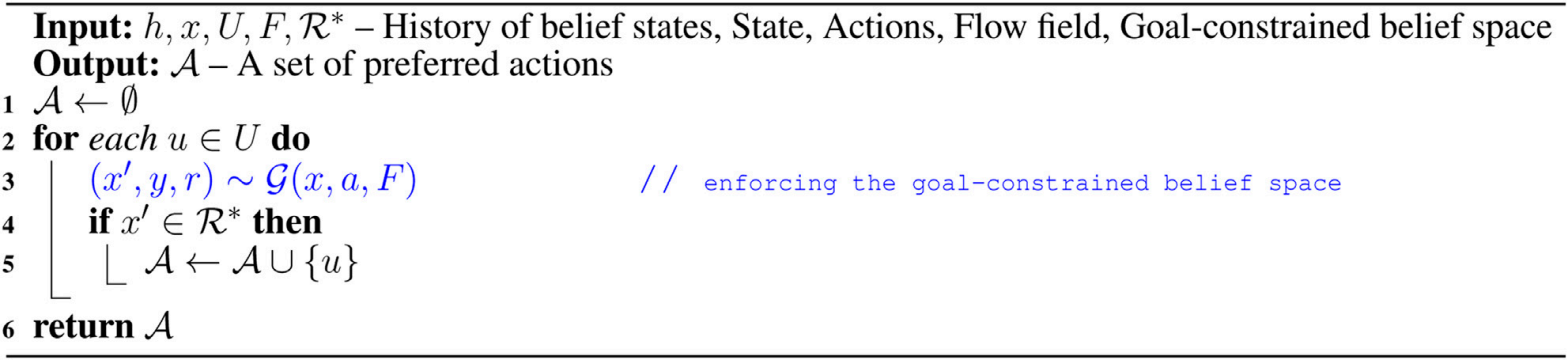

**Algorithm 2:** Search ($h,F,{ℛ}^{\text{*}}$).
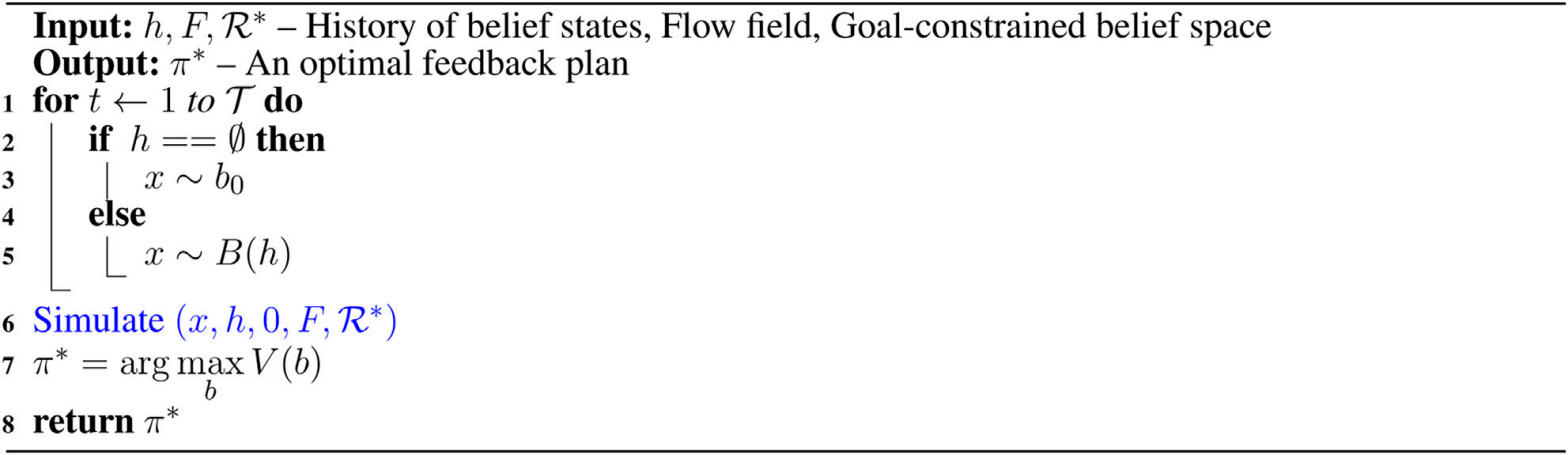

Algorithm 3 simulates an action and keeps track of its outcome. We refer to a complete simulated trial as a rollout where we keep track of actions and their outcomes as history *h*. To plan with energy-awareness, we incorporate the ocean dynamic model *F* in Algorithm 4 as a prior to the simulator G. Therefore, during a rollout, the set of available preferred actions and their outcomes take advantage of the prior knowledge. In Algorithm 4, we compute the reward values of actions by considering the flow field. The reward value is calculated high when a simulated action takes advantage of the flow field. Otherwise, the reward value is calculated low. For instance, if the vehicle simulates a particular action in a rollout, using transition probabilities and the ocean dynamic model, we first generate a simulated trajectory and then evaluate the trajectory with respect to the goal location. To evaluate a simulated trajectory, we employ the particle filter, where each state on the trajectory is considered as a particle and the goal location can be thought of as a landmark (see this work (Kim et al., 2019) for a detailed explanation of particle filter in the robot localization). When considering the next step of this rollout, the LRAUV knows which action from the set of available actions is more likely to drive it to the goal location by computing the reward associated with each action.

**Algorithm 3:** Rollout (x,h,β,F).
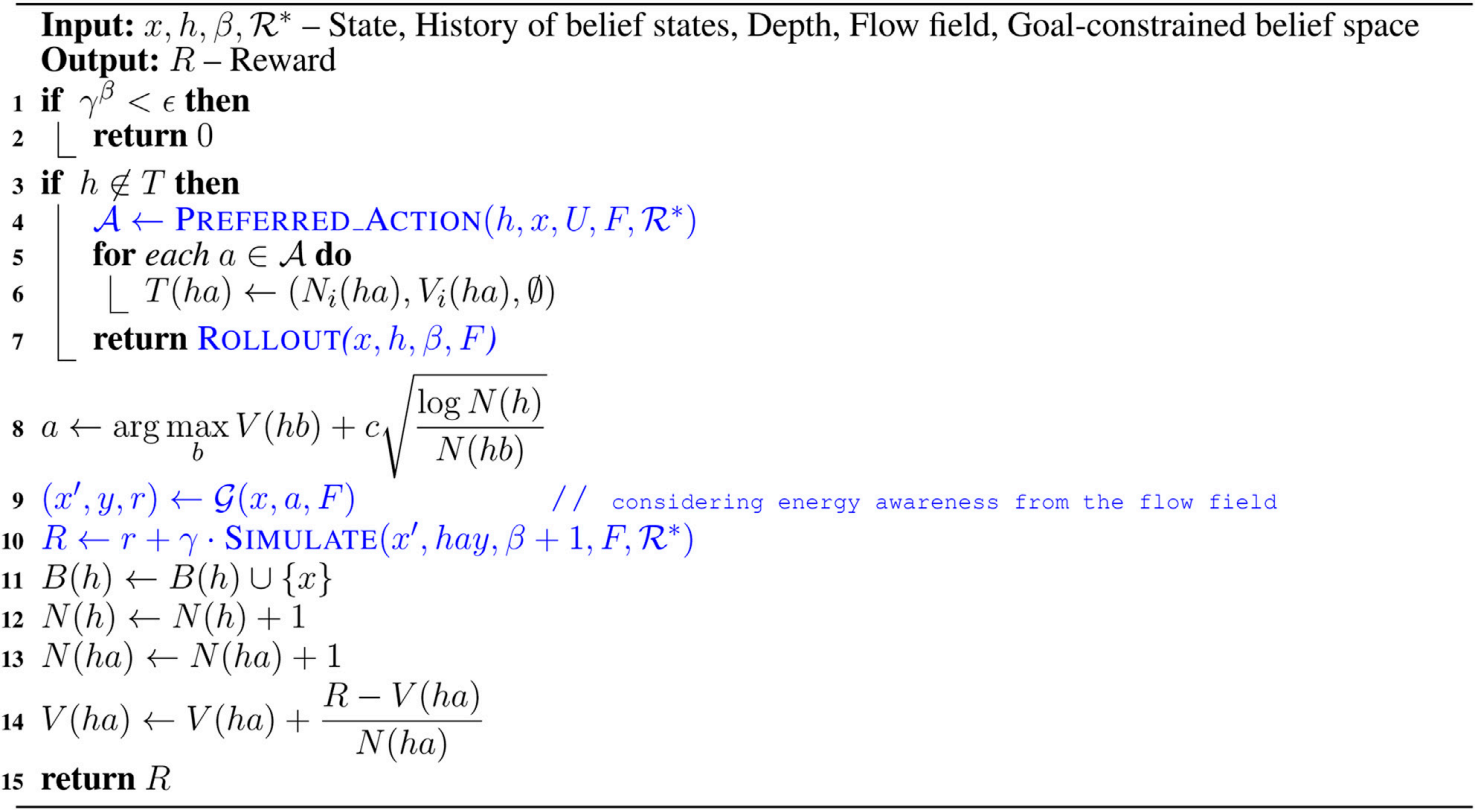

**Algorithm 4:** Preferred_Action ($h,x,U,F,{ℛ}^{\text{*}}$).
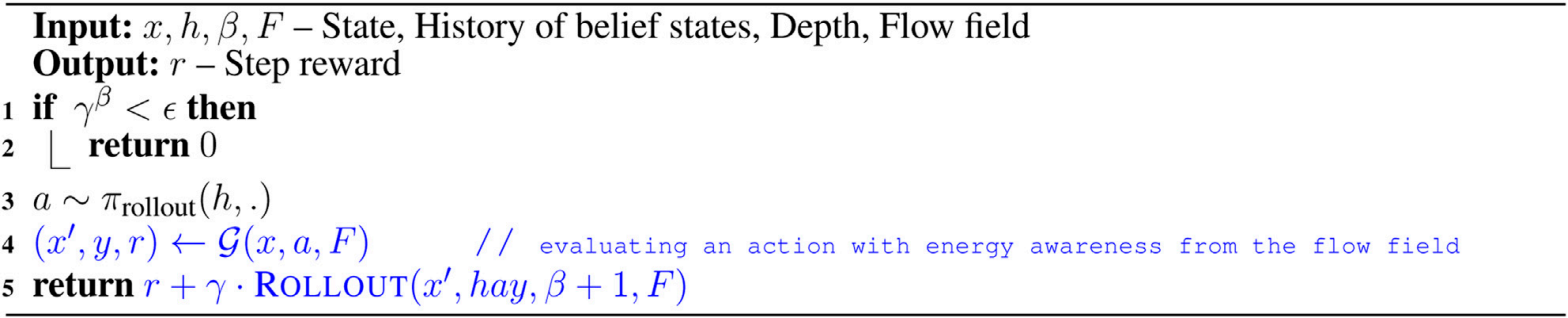

## 5 Experimental Results

In this section, we examine a Tethys-like LRAUV’s kinematic model and evaluate its navigation solution in an underwater environment under motion and sensing uncertainties. The experiments are conducted on a Unix/Linux computer with Intel Core i7 4.5 GHz processor and 32 GB memory.

### 5.1 LRAUV Kinematic Model

The vehicle motion is noisy due to the inherent dynamic nature of water flow of the underwater environment. The vehicle observation model suffers uncertainty in measuring distances and locations in sensor-denied, such as GPS, underwater environments. We modeled our vehicle motion and observation models under Gaussian noise. This setup also makes our Tethys navigation problem a POMDP problem.

Let $E_{l}\in {\mathbb{R}}^{n}$ be the state space of a water current layer $W_{l}$ and $U\in {\mathbb{R}}^{m}$ be the action space of the vehicle, where m≤n. Let $Y\in {\mathbb{R}}^{p}$ be the observation space of the vehicle sensors. The state transition model of our vehicle similar to a unicycle-model can be written as

$$x_{t+1}=x_{t}+u_{t}\cos\left(\theta_{t}\right),$$ (8)

$$y_{t+1}=y_{t}+u_{t}\sin\left(\theta_{t}\right),$$ (9)

$${\dot{\theta }}_{t}=\omega_{t}.$$ (10)

We incorporate water flow fields as prior knowledge in our motion model for the vehicle. In other words, the next transition state of the vehicle is influenced by the water flow field of a current layer as well as its actions. The unicycle motion and observation models for the vehicle can be expressed as

$$\dot{x}=f\left(x_{t},u_{t},d_{t}\right)=A_{t}x_{t}+B_{t}u_{t}+d_{t}d_{t}∼N\left(0,D_{t}\right),$$ (11)

$$\dot{y}=h\left(x_{t},w_{t}\right)=C_{t}y_{t}+w_{t}w_{t}∼N\left(0,W_{t}\right),$$ (12)

in which A is the state transition matrix of dimension n×n, B is the action transition matrix of dimension n×m, C is the sensor observation matrix of dimension p×n, and $d_{t}$ and $w_{t}$ represent the motion and sensor noise from a zero-mean Gaussian with variance $D_{t}$ and $W_{t}$ respectively.

The importance of incorporating water flow fields as the ocean dynamics in our motion model is that a Tethys-like vehicle is deployed to navigate through the water flow. However, the vehicle can leverage pressure, velocity, and acceleration of flow fields at times to perform a drifting action and save energy in its long-term mission. It is also important to note that motion and sensor noises provide motion and observation uncertainties but flow fields can be utilized for performing a passive action (drift) with no actuation and thus saving energy.

The updated observation model with energy awareness from the ocean dynamics can be expressed as

$$\dot{y}=h\left(x_{t},w_{t}\right)=C_{t}y_{t}+w_{t}+D\tilde{u},$$ (13)

in which the energy awareness $\tilde{u}=\left[\phi ,\psi \right]$ and its weight $D=\text{diag}\left(k_{u},k_{w}\right)$, where $k_{u},k_{w}>0$. The energy awareness for a specific location *q* on the water current is expressed as

ψ=arctan(ν(q),μ(q)), (14)

$$\phi =\tanh\left(x^{2}+y^{2}\right),$$ (15)

where ϕ is the angular velocity and ψ is the linear velocity of the flow field.

### 5.2 Simulation Results

A simulated Tethys-like LRAUV with the above kinematics model can take nine actions that include actions toward eight compass directions, *i.e.,* N, NE, E, SE, S, SW, W, NW along with drift (idle). The task for the vehicle is to reach a designated goal state with an energy-aware trajectory by utilizing water currents as much as possible. In our simulation, when LRAUV takes an action, the outcome of that action could be any of three observations, *i.e.,* goal, intermediate, and outside.

To incorporate the water flow pattern in our simulation, we used the ROMS (Shchepetkin and McWilliams, 2005) predicted ocean current data observed in the SCB region. The 3-D ocean environment was taken into account as a simulated environment for the Tethys movements having six 2-D ocean surfaces at six different water current layers or depths (e.g., 0 m, 5 m, 10 m, 15 m, 20 m, and 25 m). Each 2-D ocean current layer is tessellated into a grid map. Each tessellated water current layer is a 21  ×  29 grip map with a spatial resolution of 1 km × 1 km.

The feedback plan synthesis using the MCTS algorithm depends not only on the distance between initial and goal locations but also on the ocean dynamics. In our experiments during the rollout step of the MCTS algorithm, we use 50 trials for each action over an approximated belief state. We then employ the particle filter to evaluate the rollout outcomes with respect to the goal location. When selecting the next best action using Algorithm 3, we utilize a simple PID controller to follow the high-level action.

**FIGURE 5:**
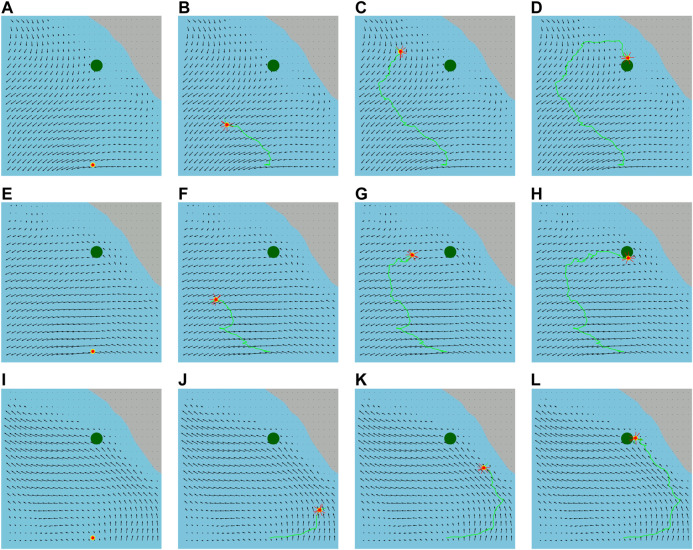
Executed trajectories delineated with the green lines of the vehicle (red circle) from its initial location to the goal location (green circle) applying the synthesized feedback plans for the first water current layer (surface layer) in **(A)–(D)** and for the third water current layer in **(E)–(H)** and for the sixth water current layer in **(I)–(L)**. The red lines around the vehicle represent a set of preferred actions of a belief state.

We implement our energy-aware feedback planning algorithm for many water current layers from our ROMS ocean current prediction data. We obtain a set of feedback plans as an output from our layer-wise feedback plan synthesis. Figure 5 illustrates the executed trajectories of the vehicle applying the synthesized feedback plans for the same pair of given initial and goal locations. For these experiments, we use longitude and latitude coordinates to represent the vehicle locations. We first set the vehicle’s initial location at (−117.84,33.54) and the vehicle needs to reach within 1 km radius of the goal location (−118.22,33.54). We then show the results for the different water current layers subject to time-varying ocean currents taking 3 h of water currents into account. A couple of videos related to these experiments can be found at https://youtu.be/FEk6QghDwgI and at https://youtu.be/9dnCam8JFTg. Table 1 shows the execution statistics of our synthesized feedback plans in terms of trajectory lengths and plan synthesis times. We assume that our vehicle operates at a constant velocity of 4.5 km/h.

**TABLE 1:** Comparison of executed trajectory lengths using synthesized feedback plans for several water current layers along with plan synthesis times for a number of hours.

Water current layer	Hour	Initial location (longitude, latitude)	Goal location (longitude, latitude)	Trajectory length (km)	Plan synthesis time (s)
2	1	(−117.84, 33.54)	(−118.22, 33.54)	4.33	0.61
	2	(−117.84, 33.54)	(−118.22, 33.54)	4.05	0.49
4	1	(−117.84, 33.54)	(−118.22, 33.54)	4.06	0.36
	3	(−117.84, 33.54)	(−118.22, 33.54)	3.44	0.42
6	1	(−117.84, 33.54)	(−118.22, 33.54)	4.38	0.41
	2	(−117.84, 33.54)	(−118.22, 33.54)	4.38	0.45

**FIGURE 6:**
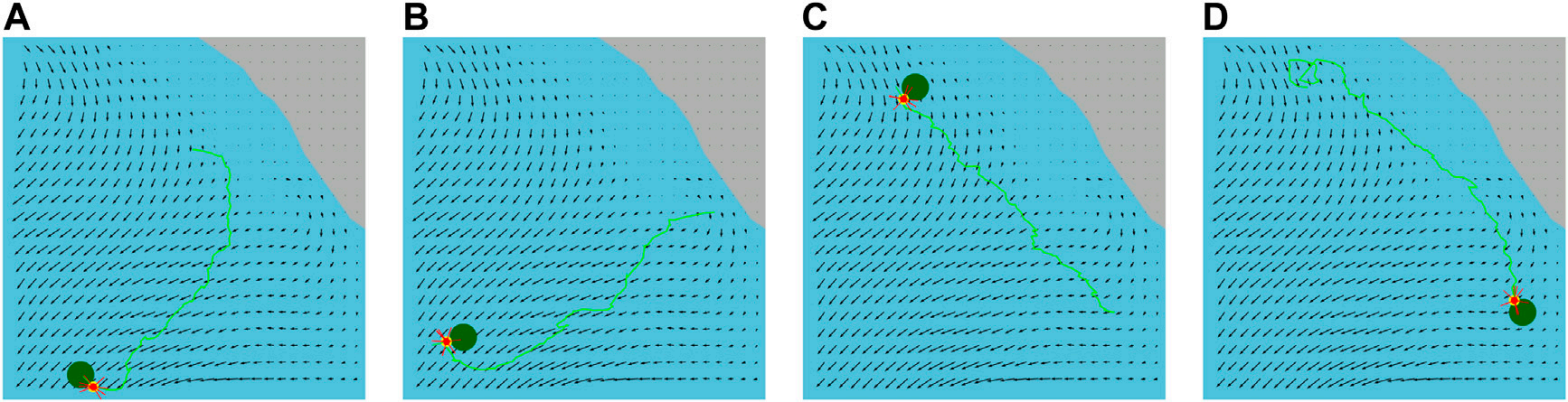
Executed trajectories (green lines) of the vehicle (red circles) from its varying initial locations to different goal locations (green circles) applying the synthesized feedback plan on the water surface layer.

We also execute trajectories applying the synthesized feedback plan for the same water current layer for the varying pairs of initial and goal locations that are illustrated in Figure 6. We observe that the trajectories of our feedback plans are not straight lines. This is because our energy-aware feedback plan chooses an action using the ocean dynamics in Algorithm 4. Therefore, the actions are selected to facilitate drifting through water currents, as mentioned in Section 4.3.

## 6 Conclusion and Discussion

This article presents an energy-aware feedback planning method for an LRAUV utilizing its kinematic model in an underwater environment under motion and sensor uncertainties. First, we generated flow fields for several water current layers from a concatenated ROMS ocean current prediction data to introduce the ocean dynamic model. Our method then synthesizes energy and computationally efficient feedback plans on goal-constrained belief spaces for many water current layers using the ocean dynamic model and sampling. Our simulation results of the execution of synthesized feedback plans demonstrated our method’s practical and potential application. There are several exciting directions to follow up on this research.

Our POMDP solution uses nine actions (eight neighboring cells and drift) for planning, which fits the scales of the ROMS resolutions (kilometers) and allows us to treat the LRAUV as a unicycle vehicle. We believe that our method can be easily generalized to incorporate modeling AUV dynamics in shorter spatial scales. We are currently using our planner, but a realistic AUV simulator (Manhães et al., 2016), could be used as a black box to generate the next states. Paring our planner with a physically realistic simulation will help us avoid complicated system identification issues and extend our methodology’s range of applications. Additionally, we would like to incorporate an initial amount of available energy, the actuator efficiency, and the drag effect in our energy model.

One desirable feature of AUV deployments in many scenarios is avoiding constant resurfacing due to energy, *stealth*, and collision safety constraints. The vehicle can collide with ships and jeopardize its mission. We are currently extending our framework to incorporate dynamic obstacles on the surface, representing, for example, boats and other vessels. We are interested in the short term to generalize this idea to other external motion fields that can be used by autonomous vehicles to use their resources efficiently. Aerial platforms such as blimps and balloons (Das et al., 2003; Wolf et al., 2010) can provide another exciting study case for our ideas.

## Data Availability

The original contributions presented in the study are included in the article/Supplementary Material, further inquiries can be directed to the corresponding author.
